# Micro-Electromechanical Acoustic Resonator Coated with Polyethyleneimine Nanofibers for the Detection of Formaldehyde Vapor

**DOI:** 10.3390/mi9020062

**Published:** 2018-02-01

**Authors:** Da Chen, Lei Yang, Wenhua Yu, Maozeng Wu, Wei Wang, Hongfei Wang

**Affiliations:** 1 State Key Laboratory of Mining Disaster Prevention and Control Co-founded by Shandong Province and the Ministry of Science and Technology, Shandong University of Science and Technology, Qingdao 266590, China; 2 College of Electronics, Communications, and Physics, Shandong University of Science and Technology, Qingdao 266590, China; ylei1994@163.com (L.Y.); 17860756595@163.com (W.Y.); wumaozeng1993@163.com (M.W.); skdwangwei1zqf@163.com (W.W.); phywjj@163.com (H.W.)

**Keywords:** film bulk acoustic resonator, formaldehyde, gas sensor, nanofibers

## Abstract

We demonstrate a promising strategy to combine the micro-electromechanical film bulk acoustic resonator and the nanostructured sensitive fibers for the detection of low-concentration formaldehyde vapor. The polyethyleneimine nanofibers were directly deposited on the resonator surface by a simple electrospinning method. The film bulk acoustic resonator working at 4.4 GHz acted as a sensitive mass loading platform and the three-dimensional structure of nanofibers provided a large specific surface area for vapor adsorption and diffusion. The ultra-small mass change induced by the absorption of formaldehyde molecules onto the amine groups in polyethyleneimine was detected by measuring the frequency downshift of the film bulk acoustic resonator. The proposed sensor exhibits a fast, reversible and linear response towards formaldehyde vapor with an excellent selectivity. The gas sensitivity and the detection limit were 1.216 kHz/ppb and 37 ppb, respectively. The study offers a great potential for developing sensitive, fast-response and portable sensors for the detection of indoor air pollutions.

## 1. Introduction

Formaldehyde, usually derived from household materials, is one of the most common indoor air pollutants. There is a strong demand for a sensitive, fast-response and portable method to detect formaldehyde for indoor environmental monitoring due to its high carcinogenicity [1]. The traditional spectroscopy, chromatography and mass spectrometry are very hypersensitive, accurate and reliable, but they are limited by the large-scale equipment, professional operation and unable to detect formaldehyde at a customer’s home [2]. The solution leads to the development of smart formaldehyde sensors with small device size and rapid response speed. So far, metal oxide semiconductor (MOS) [3,4,5], carbon nanotubes (CNTs) [6,7,8], conductive polymer [9] have been used to fabricate formaldehyde sensors based on field effect, resistive and electroacoustic principles. Over the past decade, the technical progress in micro-electromechanical systems (MEMS) brings a novel development direction for the microsensors. 

Film bulk acoustic resonator (FBAR) is a promising microelectromechanical system (MEMS) resonator and has obtained the preliminary success in radio frequency communication technologies [10,11,12]. Moreover, its applications for gas [13,14,15,16] and biochemical detections [17,18,19,20] have received attention thanks to the high sensitivity and micron-scale size. Compared with the conventional electroacoustic resonator such as quartz crystal microbalance (QCM), the important advance of FBAR is the use of 1–2 microns-thick piezoelectric films to replace the crystal plates, which provides a fundamental working frequency at several gigahertz and enough mass sensitivity to probe a single gas molecules layer [21,22]. In addition, FBAR is fabricated by standard MEMS process, thereby realizing the ability to inexpensively combine a number of sensors on a chip and integrate them with the analytical circuits. For gas-sensing applications, the FBAR usually works as a mass-loading platform. A sensitive layer is coated on the device surface to absorb the target molecules. The small additional mass on the sensing layer is detected by monitoring the variation of resonant frequency. As a result, the properties of sensitive coating determine the molecule recognitions, and directly affect the sensitivity, stability and reversibility of the sensor. Up to now, a variety of sensitive coatings, such as polymers [23,24], proteins [25,26,27], aptamers [28,29,30,31], enzymes [32,33], supramolecular monolayers [34,35], hydrophilic film [23] and CNTs [36] have been employed for FBAR sensors to achieve the selectivity for different analytes.

Polyethyleneimine (PEI) and its derivatives are regarded as an appropriate formaldehyde-sensitive material since PEI can efficiently adsorb formaldehyde molecules via a reversible reaction of primary amines. Therefore, polystyrene (PS)/PEI [37], poly(vinyl alcohol) (PVA)/PEI [38], TiO_2_/PEI [39], and CNTs/PEI composites [40] have been reported as the sensitive coating of mass-loading sensors for the detection of formaldehyde vapor. On the other hand, in the view of the high sensitivity of FBAR devices, the nanostructured materials featured with high surface areas and numerous sites are the potential coating for gas sensing. 

In this paper, we developed a promising strategy to combine PEI nanofibers and the FBAR with high mass sensitivity to construct a formaldehyde microsensor. The PEI nanofibers were directly deposited on an AlN FBAR surface by a simple electrospinning method. Benefiting from the high working frequency at 4.4 GHz, the proposed FBAR was able to measure the ultra-small mass change produced by the interaction between formaldehyde molecules and the PEI nanofibers with linear response characteristics, fast response/recovery rate and excellent selectivity.

## 2. Device Configuration and Fabrication

### 2.1. Schematic Structure and Sensing Mechanism 

**Figure 1:**
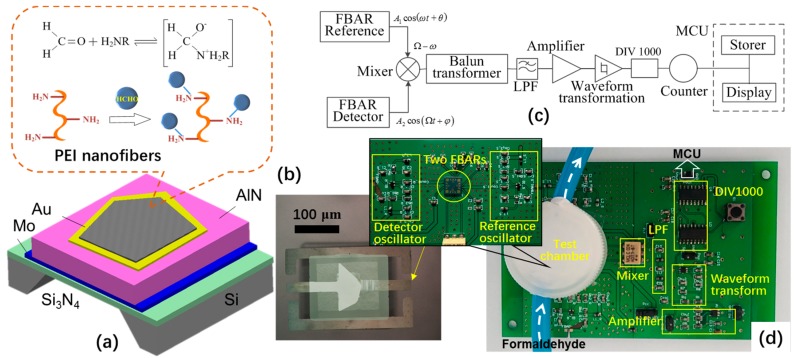
(**a**) Schematic structure and sensing mechanism of the film bulk acoustic resonator (FBAR) sensor coated with polyethyleneimine (PEI) nanofibers; (**b**) Photomicrograph of the fabricated device; (**c**) Block diagram of the testing system based on differential frequency processing; (**d**) Completed circuit board of the testing system.

Figure 1a,b shows the schematic structure, sensing mechanism and the photomicrograph of the FBAR formaldehyde sensor. The major structure of FBAR (300 × 150 μm^2^) is a sandwiched Au (100 nm)/AlN (1 μm)/Mo (100 nm) piezoelectric stack built on a Si_3_N_4_ sputtered layer (0.6 μm). The PEI nanofibers were deposited on the surface of top Au electrode as the specific sensitive coating. The active resonance and sensing area are overlapped between the two electrodes (3296 μm^2^). When the FBAR sensor is exposed to gaseous formaldehyde, the reversible nucleophilic addition reaction happens between the amines of PEI and the vapor molecules at room temperature [41]. In fact, there is a σ-bond and a π-bond in the formaldehyde molecule. Because of the difference in electron affinity, the oxygen atom side shows electronegativity while the positive side of carbon atoms can be considered as the electrophile. In the amine group, the lone pair in the nitrogen atoms works as the nucleophile and engages in the reaction with the π-bond in formaldehyde molecules. Therefore, the vapor absorption can be measured by monitoring the downshift of resonant frequency based on the mass-sensitive mechanism.

In order to minimize the environmental disturbance, the practical differential frequency method was used to extract the sensing response as shown in Figure 1c. For this purpose, both the FBAR coated with PEI nanofibers and the reference FBAR device (without coating) were wire-bonded to a printed circular board (PCB) and packaged in the test chamber (Figure 1d). Upon the exposure of formaldehyde, the resonant frequency of the former device decreased while the reference frequency measured from the latter kept stable. The frequency difference between the two devices was read out by a testing circuit as the sensing response to formaldehyde vapor. All components in the test circuit were off-the-shelf. At first, the two FBAR devices were driven by independent Colpitts oscillators. The frequency signals were put into the mixer and then passed through the balun transformer, low-pass filtered, the amplified, waveform converter and frequency dividing circuits. At last, a micro controller unit was used to count, read out and store the differential frequency. The details of the circuit design were shown in Supplementary Material (Figure S1).

### 2.2. Fabrication of Film Bulk Acoustic Resonator (FBAR) Device 

**Figure 2:**
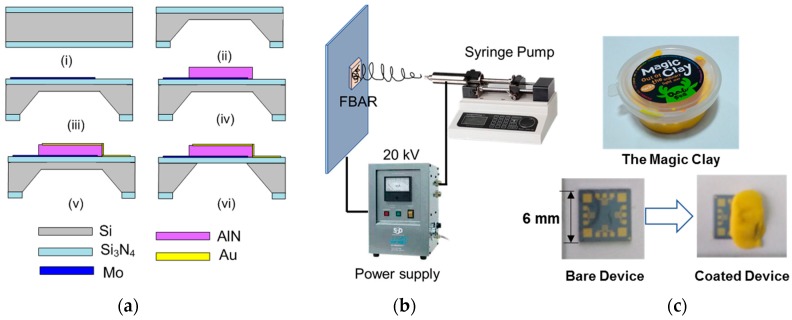
(**a**) Fabrication process of the FBAR formaldehyde sensor: (i) Growth of Si3N4 film; (ii) Etching of one side of the silicon wafer; (iii) Deposition and pattern of bottom Mo electrode; (iv) Deposition of AlN film; (v) Preparation of top Au electrode; (vi) Dry etching of the residual silicon. (**b**) Schematic diagram illustrating the electrospinning deposition of PEI nanofibers. (**c**) Photographs of the FBAR devices before and after the coating of clay.

The FBAR sensor was fabricated with a four-mask process using standard MEMS technology as shown in Figure 2a. First, a low-stress Si_3_N_4_ layer was grown on both sides of a (100) silicon wafer by low-pressure chemical vapor deposition. Then, an initial cavity was formed on one side of the wafer by wet etching (80 °C KOH) with the patterned Si_3_N_4_ layer as the mask. About 15-μm-thick silicon was left to support the following process on the other side of the wafer. Next, the Mo/AlN/Au piezoelectric stack was prepared by radio frequency magnetron sputtering and patterned by conventional photolithography technique. Finally, the residual silicon under the stack was etched by deep reactive ion etching to isolate the resonator acoustically from the substrate.

### 2.3. Electrospinning Deposition of Polyethyleneimine (PEI) Nanofibers

After the fabrication process, the silicon wafer was cut into small pieces with the area of 6 mm × 6 mm (as shown in Figure 2c). Two FBAR devices on the opposite side were used in this experiment as the detector and reference device, respectively. The reference device without sensitive layer was manually coated with kid’s magic clay (DoDoLu, Zhigao colored clay Co., Ltd., Jinhua, China) before the electrospinning process (see Figure 2c). The main components of magic clay are polyethylene ethanol, cross-linking agent and water. The viscous magic clay was attached to the device surface and then was easily removed after natural drying without any damage on the device structure. The PEI aqueous solution (10 wt %, MW = 25,000, Macklin Biochemical Co., Ltd., Shanghai, China) was used as the electrospinning solution. The feed rate of the solutions was regulated at 5 mL/h by a syringe pump (LSP02, Longer Precision Pump Co., Ltd., Baoding, China). By applying the voltage of 20 kV between the syringe and the conductive sheet at a tip-to-collector distance of 20 cm, the nanofibers were continuously deposited on the FBAR device surface. After electrospinning, the device was dried at 40 °C in vacuum for 30 min to evaporate the solvent. In addition, a flat PEI film was spin-coated (30 wt % PEI solution, 2500 rpm for 45 s) on the surface of another FBAR device to compare the formaldehyde sensing characteristics.

## 3. Results and Discussion

### 3.1. Characterization of the Resonator

**Figure 3:**
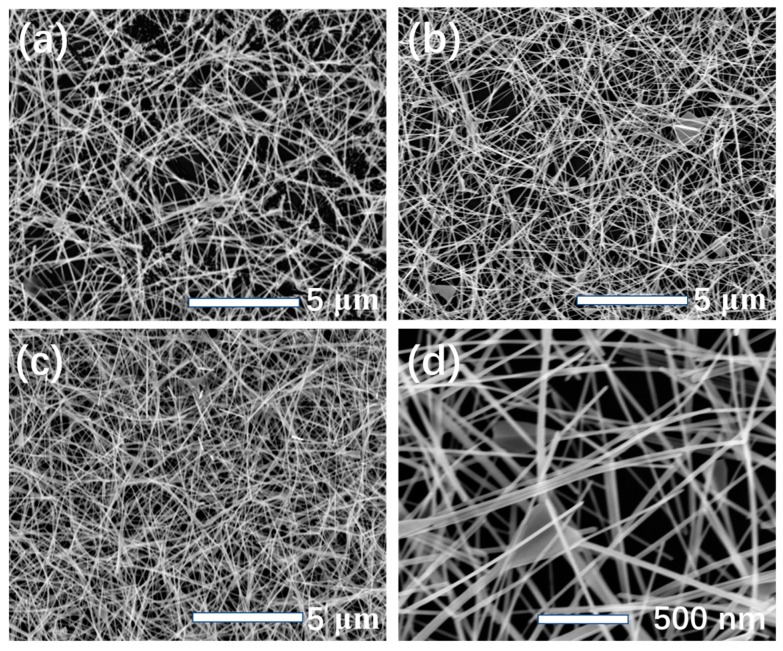
Typical FE-SEM images of the PEI nanofibers on the FBAR surface for the deposition times of (**a**) 50 s, (**b**) 150 s and (**c**) 300 s. The detail view of the nanofibers is shown in (**d**).

The surface morphology of the sensitive coating on the FBAR surface was observed by a field emission scanning electron microscope (FE-SEM, S-4800 Hitachi, Tokyo, Japan) as shown in Figure 3. The average diameter of the PEI fibers was about 40 nm with random orientations. The distribution became denser with the increase of deposition time, which had an obvious effect on the formaldehyde response. Compared with the layer imbedded with nanoparticles [39] or nanotube [40] prepared by spin coating, the electrospinning method forms the three-dimensional structure, which can provide a larger specific surface area for vapor adsorption and diffusion. 

**Figure 4:**
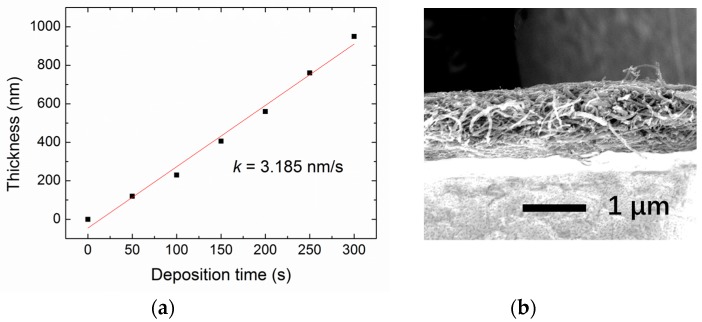
(**a**) Dependence of the thickness of the PEI nanofiber layer on the deposition time; (**b**) Typical cross-view SEM image of the PEI nanofiber layer with the deposition times of 300 s.

**Figure 5:**
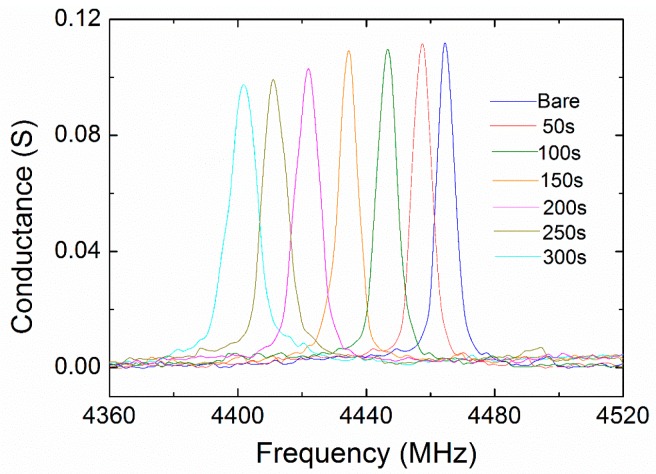
The admittance curves of the bare FBAR device and PEI nanofibers-coated FBAR devices. The times of electrospinning deposition were 50–300 s.

**Figure 6:**
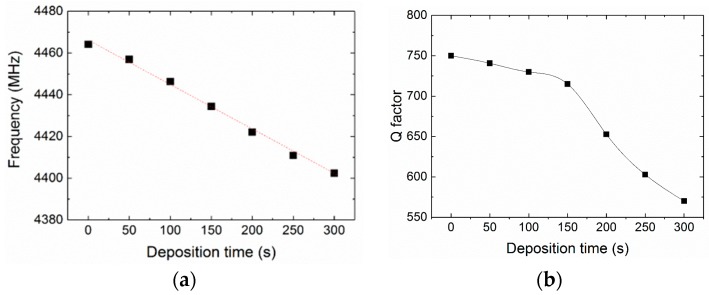
The changes of (**a**) resonant frequency and (**b**) Q factor of the PEI nanofibers-coated FBAR devices for different deposition times.

The thickness of the PEI nanofiber layers was estimated by the cross-view SEM images as shown in Figure 4. The thickness of the PEI nanofiber layer exhibited a linearly dependence on the deposition time with the rate of 3.185 nm/s. Figure 5 shows the admittance of the FBAR sensors coating with different amounts of nanofibers. The device frequency linearly went down from 4464.15 MHz to 4402.41 MHz with increasing nanofibers (Figure 6a). In addition, the Q factors of the FBAR devices were determined by the Butterworth–Van Dyke model [42] to evaluate the energy loss because of the sensitive coating. As shown in Figure 6b, the coated PEI nanofibers only caused a small influence on Q factors (<5%) when the deposition time was less than 150 s. However, the loading of superfluous fibers resulted in a dramatic degradation of Q factors, which may be ascribed to the scattering and absorption of acoustic energy from the porous structure on the wave propagation path. Besides, the resonant frequency of the bare device shows a temperature coefficient of frequency (TCF) of −52.2 ppm/°C [43], which can be attributed to the thermal expansion of the piezoelectric film and the change of the acoustic velocity.

### 3.2. Effect of Deposition Time on the Formaldehyde Response

**Figure 7:**
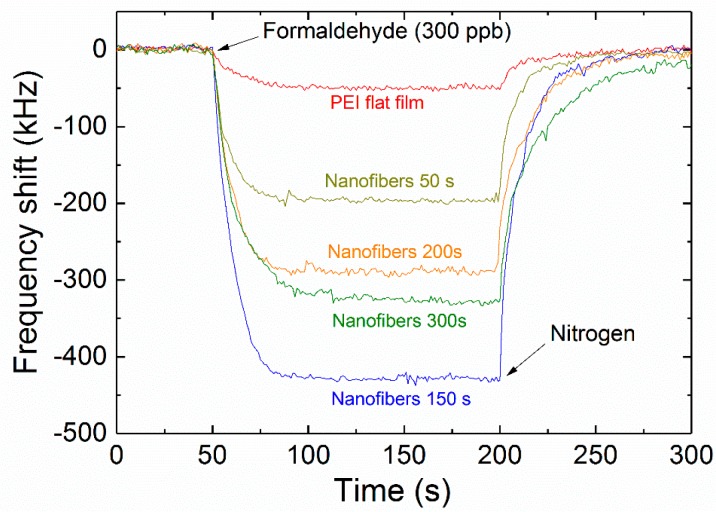
Time-dependent frequency response of the FBAR sensors coated with PEI flat film and different amounts of PEI nanofibers when exposed to 300 ppb formaldehyde vapor. All the tests were performed at room temperature and 40% relative humidity.

In the gas-sensing tests, the formaldehyde samples with required concentration were obtained by multiple dilution of the standard vapor (Changyuan Gas, Nanjing, China). All the gas tests were performed at the constant temperature condition (22 °C). Figure 7 shows the time-dependent frequency response of the FBAR sensors coated with flat PEI film and different amounts of PEI nanofibers in the formaldehyde vapor with the concentration of 300 ppb. Every sensing cycle comprised both absorption and desorption processes. At first, nitrogen was delivered to the test chamber and the stable resonant frequency was recorded as the baseline. After the formaldehyde was injected, the formaldehyde molecules were absorbed on the sensitive coating resulting in the downshift of resonant frequency. When the nitrogen was introduced again, the formaldehyde molecules were blown off from the sensitive coating and thus the response gradually raised to the baseline. 

The structure and amount of the sensitive coating had an obvious influence on the absorption/desorption behavior of formaldehyde. At the formaldehyde concentration of 300 ppb, the response of the flat film coated sensor was very small (~50 kHz). As expected, the fibrous structure could absorb more molecules and significantly enhance the sensitivity. The adsorption of formaldehyde onto the sensitive coating is maintained by the polymeric amines in PEI via the reversible nucleophilic addition reaction to form Schiff base [38]. As shown in the SEM images, the PEI fibers were connected to each other, forming net-like scaffolds, which increased the surface area and absorption sites and allowed the formaldehyde molecules to diffuse more easily inside the large space to react with the amine groups of PEI.

In particular, the sensor with the appropriate deposition time (150 s) exhibits the highest frequency downshift (~430 kHz) among the devices. Some references considered the coupled resonance between the microstructure coating and acoustic wave sensor [44,45,46,47]. However, in our study, the frequency shows a linearly negative shift with increasing of deposition time. The “drop and jump” change (the hallmark feature of coupled resonance) was not observed in Figure 6. On the other hand, the theoretical model proposed by Mansfeld [48] indicates that the mass-loading sensitivity increases sharply when the thickness of sensitive layer is close to a quarter of the acoustic wavelength. The Young modulus and acoustic velocity of solid-state PEI is 3.1–3.7 GPa and 2100–2300 m/s, respectively [49]. For the FBAR working frequency at 4.4 GHz, the corresponding quarter of the acoustic wavelength is 470~520 nm, which is very close to the thickness at the deposition time of 150 s in consideration of the velocity deviation between the solid state and nanofibers.

### 3.3. Sensitive Performance of the Optimized Sensor

**Figure 8:**
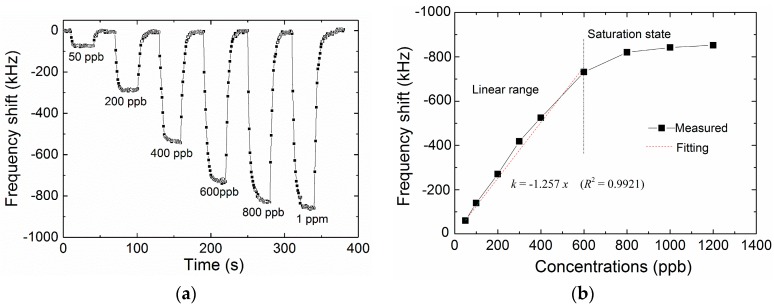
(**a**) Dynamic measurement of the FBAR sensor coated with PEI nanofibers exposed to the formaldehyde pulses with increasing concentrations at room temperature and 40% relative humidity; (**b**) Dependence of the frequency shift on the formaldehyde concentration.

On the basis of the above results, the optimized deposition time of 150 s was selected for further tests. Figure 8a shows the dynamic measurement of the optimized FBAR sensor exposed to the formaldehyde pulses with increasing concentrations. The fast adsorption and complete desorption took place with the response time of 10–25 s and the recovery time within 60 s. A saturated response was observed at vapor concentrations higher than 600 ppb, which is associated with the saturable adsorption of the PEI nanofibers. However, this detectable range could be accepted for the indoor air monitoring because serious throat and nasal irritation will occur when the formaldehyde concentration reaches about 1 ppm. As shown in Figure 8b, the frequency downshift of FBAR sensor was linearly proportional to the formaldehyde concentration before saturation with the regression coefficient (R^2^) of 0.9921. The gas sensitivity, defined as the slope of calibration curve, was estimated to be −1.257 kHz/ppb over the linear region. The limit of detection (LOD) is given by LOD = 3*σ*/*S*, where *S* is the gas sensitivity of the sensor and σ is the average noise of the intrinsic frequency [50], respectively. Benefiting from the high mass-sensitivity and the porous structure of PEI fibrous coating, the LOD of the FBAR sensor was as low as 37 ppb (*σ* ≈ 15 kHz). In comparison, the formaldehyde detection limit of a recently reported QCM sensor is 600 ppb [40]. The sensor noise determines the minimum detectable frequency shift and thus is related to the detection limit. In this study, the average noise level was about 15 kHz at the resonant frequency of 4.4 GHz, which is higher than that of commercialized QCM systems (typically 0.01 Hz/8 MHz). Even so, the FBAR sensor still exhibits a very high sensitivity and low detection limit. It is believed that the sensitivity can be further improved by optimizing the device structure and test circuit.

For the mass-loading sensors, the higher working frequency produces a larger frequency shift with the same mass change. Therefore, the FBAR sensor exhibits a gas sensitivity that was about ten times higher than that of the QCM formaldehyde sensors [37,38,39]. The LOD of FBAR sensor reached the same levels of MOS [3,4,5] and CNTs sensors [6,7,8]. Remarkably, in our case, we are able to employ the operation at room temperature and the integration capability into micro-electromechanical systems. Furthermore, compared with the analog resistance or current signals produced by MOS and CNTs sensors, the digitized frequency response used by FBAR is more robust and can be read out directly without the need of analog–digital conversion. Benefiting from the micrometer-scale size and the MEMS fabrication, an e-nose could be constructed by integrating a large number of FBAR devices with different sensitive coatings. Each sensor is sensitive to some analytes with different responses; thus, a fingerprint pattern is generated for specific target recognition from the complex environment. For example, Yao el al. [34] demonstrated an e-nose type gas sensor for the selective detection of volatile organic compounds based on the FBAR sensor array functionalized with four supramolecular monolayers (p-tert-butyl calix[8]-arene, porphine, β-cyclodextrin, and cucurbit[8]uril.).

### 3.4. Influence of Relative Humidity

**Figure 9:**
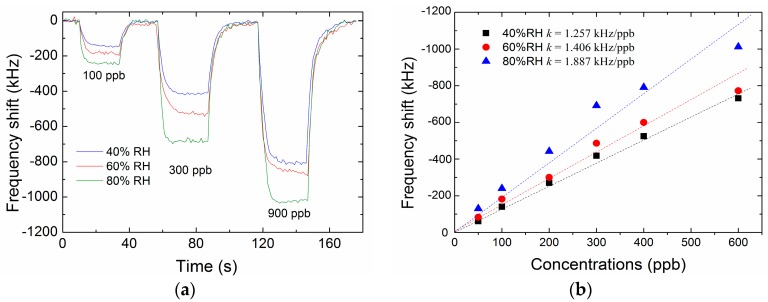
(**a**) Real-time frequency response of the optimized FBAR sensor exposed to three typical formaldehyde concentrations at the relative humidity of 40%, 60% and 80%. (**b**) Plots of frequency shift as a function of formaldehyde concentration at the relative humidity of 40%, 60% and 80%.

In order to evaluate the influence of humidity on the sensing characteristics, the formaldehyde vapor mixed with saturated water vapor was delivered to the test chamber. Figure 9a shows the frequency response of the optimized FBAR sensor in three typical vapor concentrations measured at different ambient humidity. Both the frequency shift and the gas sensitivity were obviously enhanced with the increase of humidity. A possible reason is that the formaldehyde molecules could easily attach to the water molecules via hydrogen bonds [51]. In the humid environment, more water molecules were attached on the hydrophilic amine groups of PEI, and thus the adsorption capacity of the sensitive coating was improved.

### 3.5. Selectivity of the Sensors

**Figure 10:**
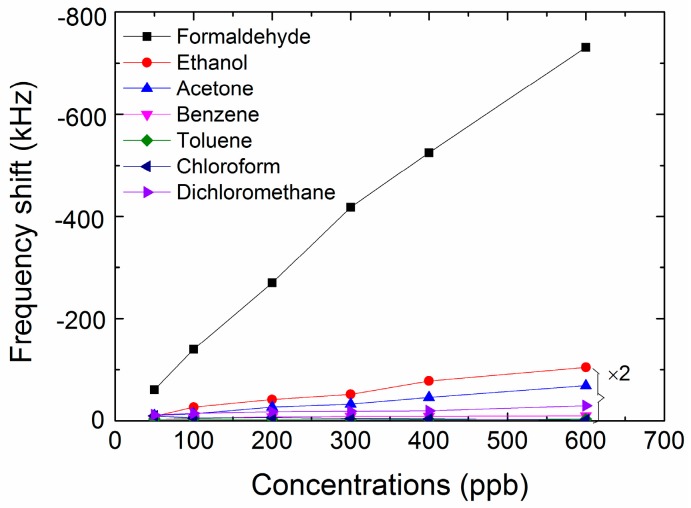
Plots of the frequency shift as a function of different concentration for formaldehyde and potential indoor pollution vapors.

The FBAR sensor was tested against several potential indoor pollution vapors, including ethanol, acetone, benzene, dichloromethane, toluene and chloroform. As shown in Figure 10, the response to formaldehyde was about ten times higher than that of ethanol (the second largest response) indicating the excellent selectivity of the FBAR sensor coated with PEI nanofibers. As discussed before in Section 2.1, the main absorbing mechanism to formaldehyde is the nucleophilic addition reaction between the carbon atoms in π-bonds (electrophile) and the nitrogen atoms in amine groups (nucleophile). As a result, the specific attachment to formaldehyde is significantly stronger than the physical or nonspecific adsorption of the interference vapors. Similar results were also observed in other PEI-based sensors [36,37,38,39].

## 4. Conclusions

In summary, we deposited PEI nanofibers on the FBAR mass-loading sensor by electrospinning and demonstrated its application for the detection of trace formaldehyde at room temperature. The interconnected PEI fibers formed a porous three-dimensional structure and provided a larger specific surface area to absorb formaldehyde molecules. The FBAR sensor exhibits a linear frequency downshift with increasing vapor concentration and excellent selectivity to formaldehyde with respect to other conventional organic vapors. The gas sensitivity is 1.216 kHz/ppb with the LOD of 37 ppb. The proposed FBAR device is a promising candidate as a mass-sensitive platform, and the studies of the electrospinning would benefit the developing of new sensitive coating for FBAR gas sensors.

## Supplementary Materials

The following are available online at http://www.mdpi.com/2072-666X/9/2/62/s1, Figure S1: Ddetails of the circuit design of the film bulk acoustic resonator (FBAR) testing system.
