# Supplementary material for: Micro-Electromechanical Acoustic Resonator Coated with Polyethyleneimine Nanofibers for the Detection of Formaldehyde Vapor
Source: Micromachines (Basel). 2018 Feb 1;9(2):62. doi: 10.3390/mi9020062 (PMC6187669; doi:10.3390/mi9020062)
Supplement: Supplementary file 1 [file micromachines-09-00062-s001.pdf]

micromachines

MDPI

Micro-Electromechanical Acoustic Resonator Coated with Polyethyleneimine Nanofibers for the Detection of Formaldehyde Vapor

Da Chen *, Lei Yang, Wenhua Yu, Maozeng Wu, Wei Wang and Hongfei Wang

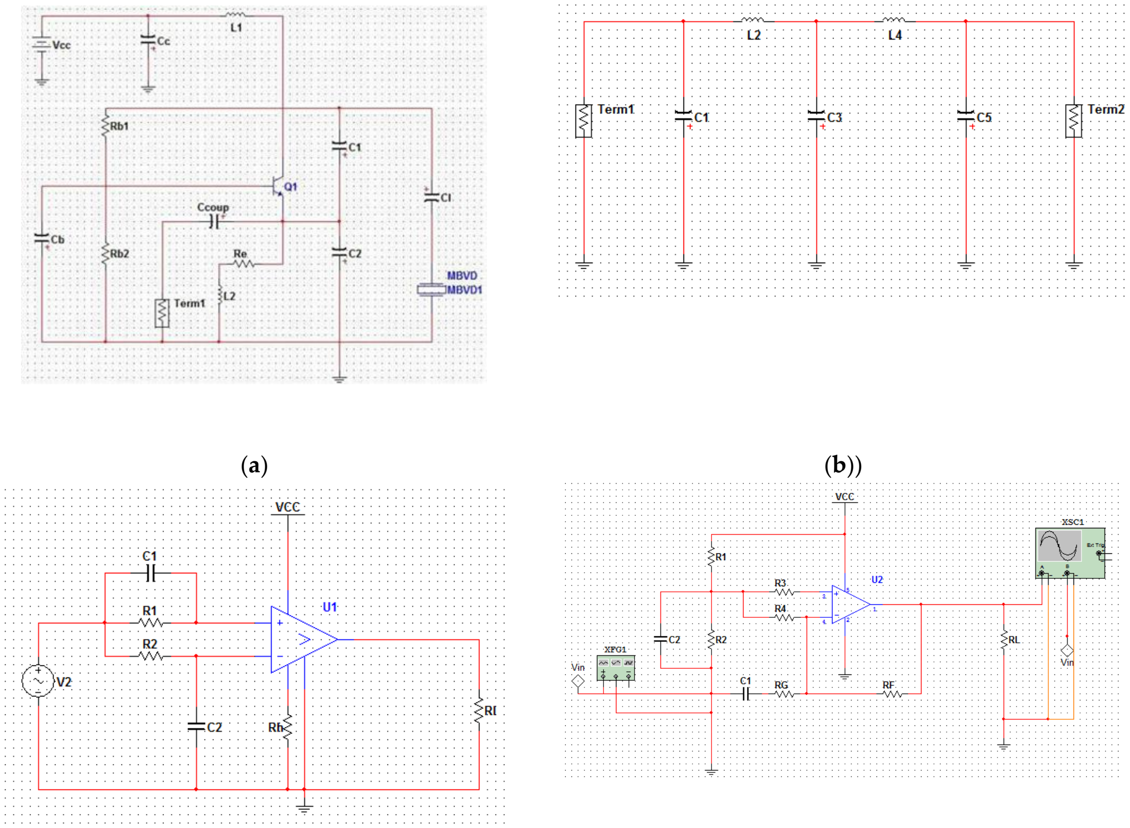

Figure S1. Ddetails of the circuit design of the film bulk acoustic resonator (FBAR) testing system. (**a**) Colpitts oscillator of the FBAR devices; (**b**) low-pass filter (LPF); (**c**) the amplifier; (**d**) the waveform transformation.
